# A metabolomics study delineating geographical location-associated primary metabolic changes in the leaves of growing tobacco plants by GC-MS and CE-MS

**DOI:** 10.1038/srep16346

**Published:** 2015-11-09

**Authors:** Yanni Zhao, Jieyu Zhao, Chunxia Zhao, Huina Zhou, Yanli Li, Junjie Zhang, Lili Li, Chunxiu Hu, Wenzheng Li, Xiaojun Peng, Xin Lu, Fucheng Lin, Guowang Xu

**Affiliations:** 1 Key Laboratory of Separation Science for Analytical Chemistry, Dalian Institute of Chemical Physics, Chinese Academy of Sciences, Dalian 116023, China; 2 State Key Laboratory of Fine Chemicals, Dalian University of Technology, Dalian 116023, China; 3 China Tobacco Gene Research Center, Zhengzhou Tobacco Research Institute of CNTC, Zhengzhou, 450001, China; 4 Yunnan Academy of Tobacco Agricultural Sciences and China Tobacco Breeding Research Center at Yunnan, Yuxi, 653100, China

## Abstract

Ecological conditions and developmental senescence significantly affect the physiological metabolism of plants, yet relatively little is known about the influence of geographical location on dynamic changes in plant leaves during growth. Pseudotargeted gas chromatography-selected ion monitoring-mass spectrometry and capillary electrophoresis-mass spectrometry were used to investigate a time course of the metabolic responses of tobacco leaves to geographical location. Principal component analysis revealed obvious metabolic discrimination between growing districts relative to cultivars. A complex carbon and nitrogen metabolic network was modulated by environmental factors during growth. When the Xuchang and Dali Districts in China were compared, the results indicated that higher rates of photosynthesis, photorespiration and respiration were utilized in Xuchang District to generate the energy and carbon skeletons needed for the biosynthesis of nitrogen-containing metabolites. The increased abundance of defense-associated metabolites generated from the shikimate-phenylpropanoid pathway in Xuchang relative to Dali was implicated in protection against stress.

Tobacco (*Nicotiana tabacum* L.) is an important model for studies on genetics, breeding, physiology and biochemistry in plant science^1^,^2^. Agronomical yield and flavor are crucial indicators of the quality of tobacco plants, which are frequently limited by the planting environment (e.g., climate and soil conditions)^2^,^3^ and by genotypes^4^. Furthermore, tobacco plants induce a series of adjustments in their metabolic and physiological functions to adapt to changes in the environment^5^,^6^,^7^. The metabolic responses of tobacco leaves to saline stress have been closely associated with treatment dosage and time. Gluconeogenesis was activated, with upregulation of sucrose, glucose and fructose, under short-term condition with low-dose salt. However, high-dose salt promoted the degradation of DNA, RNA and protein to inhibit tobacco growth under prolonged stress^5^. Enhancements in the alpha activity of tobacco leaves grown with phosphate fertilizers indicated that the reasonable use of phosphate fertilizers reduces damage from radioactivity and promotes the growth of tobacco^6^. In addition, transgenic tobacco plants overexpressing the allene oxide synthase (AOS) gene showed enhanced tolerance to zinc stress compared with wild-type samples^7^.

Metabolomics is a promising analytical technology that has been used to unravel the metabolic fluctuations of crucial plant molecules and the metabolic flux associated with various genotypes^8^,^9^ and growth environments^10^,^11^. Common analytical technologies used for metabolomics primarily include chromatography coupled with mass spectrometry^12^,^13^,^14^ and nuclear magnetic resonance spectroscopy^15^,^16^. Gas chromatography-mass spectrometry (GC-MS) has also been widely employed in metabolomics studies, due to its high quality and reproducibility, wide dynamic range, universal mass spectral library and ability to detect hydrophilic metabolites after derivatization^17^. Recently, we developed a pseudotargeted metabolomics approach based on GC-MS with selective ion monitoring (SIM), in which the characteristic ions of metabolites were identified in untargeted metabolic profiling data from quality-control samples^18^. This approach integrates the advantages of targeted and untargeted methods^2^ and has been used to study the metabolic profiles of various tissues samples^12^,^19^. In addition, capillary electrophoresis-mass spectrometry (CE-MS), as a novel and promising method with small injection volume and high resolution, has been unitized to separate and detect ionic compounds based on the different migration rates of charged metabolites^14^. Accordingly, a combination of GC-MS and CE-MS could clearly enhance the coverage of metabolites (e.g., carbohydrates, organic acids, and nucleosides)^20^.

Several studies concerning the metabolic responses of tobacco plants to the environment have been conducted, yet relatively few studies have focused on the dynamic changes of metabolic responses to geographical fluctuations during tobacco growth stages. Two major tobacco-planting regions consist of the Dali District from Yunnan province located in southwest China and the Xuchang District from Henan province located in central China. The tobacco grown in these regions possesses markedly different quality traits that reflect the geographical variation. In the present study, we investigated the impact of the planting environment (Dali and Xuchang) on tobacco leaf metabolism during the growth and senescence period (Fig. 1a,b) by using pseudotargeted GC-SIM-MS and CE-MS methods. It was obvious that both environment (planting regions) and growth (four growing periods) have great effects on the metabolic phenotype of tobacco leaves compared with tobacco cultivar. Moreover, the metabolic signatures associated with geographical origin and development stage were defined, and the effects of environment factors to complex metabolic regulatory systems were identified.

## Results

### Analytical characteristics of tobacco metabolic profiling methods

Both pseudotargeted GC-SIM-MS and CE-MS were used for tobacco metabolic profiling analysis. A double-concentration QC sample was prepared for peak identification using a pseudotargeted GC-SIM-MS method. A total of 329 peaks were acquired. Among these, 107 metabolites were verified using authentication standards, and 38 compounds were identified after matching with mass spectral libraries (e.g., Wiley, Mainlib and Fiehn). Using the CE-MS method, 148 metabolites were identified from the library search^21^; 115 and 33 metabolites were detected under cationic and anionic modes, respectively. The pseudotargeted GC-SIM-MS and CE-MS metabolic methods showed good complementarity, with only 37 metabolites in common. After the removal of duplicates and unknown metabolites using both methods, a total of 237 metabolites, which are involved in carbon metabolism (e.g., sugar metabolism, glycolysis, tricarboxylic acid (TCA) cycle and phenol metabolism) and nitrogen metabolism (e.g., amino acid metabolism, nucleotide metabolism and polyamine metabolism), were identified and reserved for further statistical analysis.

Typical chromatograms from pseudotargeted GC-SIM-MS and from CE-MS in cationic and anionic modes are shown in Supplementary Fig. S1a1, S1b1 and S1c1, respectively. To evaluate the reproducibility of pretreatment and the stability of the analytical apparatus, the QC samples were identically inserted into the analytical sequence of all samples. The QC samples were closely assembled into the principal component analysis (PCA) score plots of GC-SM-MS (Supplementary Fig. S1a2) and of CE-MS in cationic (Supplementary Fig. S1b2) and anionic modes (Supplementary Fig. S1c2). The RSD (relative standard deviation) distribution plots of all variables in the 34 QC samples showed that the RSD values of 92.45%, 98.7% and 99.65% of the total peak area for GC-SIM-MS (Supplementary Fig. S1a3) and for the cationic (Supplementary Fig. S1b3) and anionic (Supplementary Fig. S1c3) modes of CE-MS, respectively, were less than 20%. These results suggest that the metabolic profiling methods based on GC-SIM-MS and CE-MS were robust and reliable.

### Global metabolic responses to geographical locations, cultivars and growth stages

**Figure 1:**
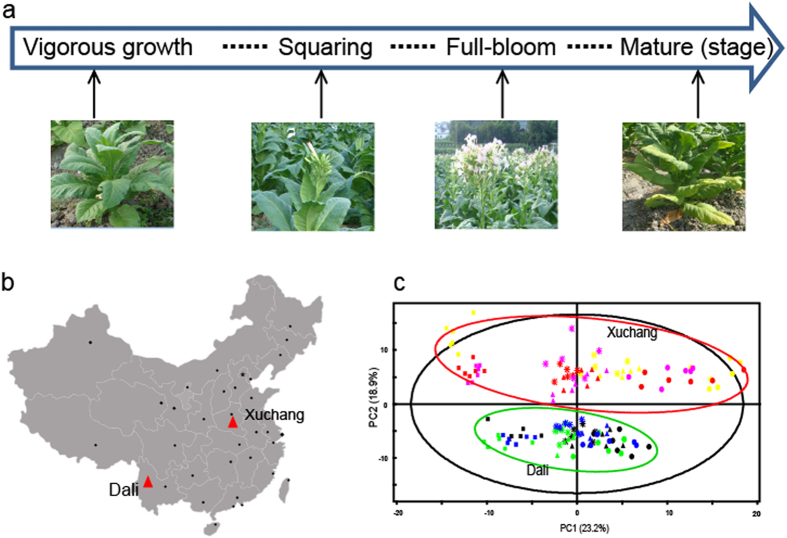
Experimental design and different metabolic profiles of tobacco leaves during growth at two different geographical locations. (**a**) Experimental design, including four developmental stages (vigorous growth, squaring, full-bloom and mature stages) of tobacco leaf development. (**b**) The geographical locations of the two tobacco plant-growing districts (Dali, Yunnan and Xuchang, Henan). (**c**) PCA analysis of three flue-cured cultivars (*cv.* HD, K326 and ZY100) from the two different growing locations during the four developmental stages. Red, purple and yellow indicate K326, ZY100 and HD, respectively, grown in Xuchang. Green, blue and black represent K326, ZY100 and HD, respectively, grown in Dali. Vigorous growth stage (○), squaring stage (∆), full-bloom stage (*), mature stage (□). The map was generated in Matlab R2014a software and modified using Adobe Illustrator CS5 software. Photos were taken by Wenzheng Li.

Dali and Xuchang are major tobacco-planting regions in China with significantly different geographical and climatic conditions (Fig. 1b and Supplementary Fig. S2). Dali is located in the southwest region of China, in a plateau monsoon climate zone that has a moderate temperature and abundant rainfall^22^. In contrast, Xuchang is a city in the Huanghuai District of China, in a warm-temperature monsoon climate zone with a high temperature and less rainfall^23^.

To investigate the global metabolic changes of tobacco in response to planting regions, cultivars and growth periods, 143 green leaves were collected from three widely used flue-cured cultivars (*cv.* K326, Zhongyan 100 (ZY100) and Hongda (HD)), grown in Xuchang, Henan and Dali, Yunnan (Supplementary Table S1). Four growth periods (vigorous growth, squaring, full-bloom and mature stage) were studied. An unsupervised PCA analysis with unit variance (UV) scaling (variables-subtracted average value divided by the standard deviation) was conducted to visualize the effects of geographical locations, cultivars and growth stages on tobacco metabolism. The model parameters (R^2^X = 0.776, Q^2^ (cum) = 0.618) indicated that 77.6% and 61.8% of the total variations are explained and predicted, respectively. An obvious separation of the tobacco leaves from the two regions was observed along the second principal component (PC2) in the PCA score plot, and the growth directions of all the tobacco leaves were identical across the first principal component (PC1) (Fig. 1c). However, no obvious differences among the three main cultivars of flue-cured tobacco (*cv.* HD, K326 and ZY100) grown in Xuchang or Dali were observed (Fig. 1c). These results show that the planting region has a greater influence than the cultivar on the metabolic phenotype of flue-cured tobacco during plant growth.

### Time dependence of metabolic alterations on geographical location

**Figure 2:**
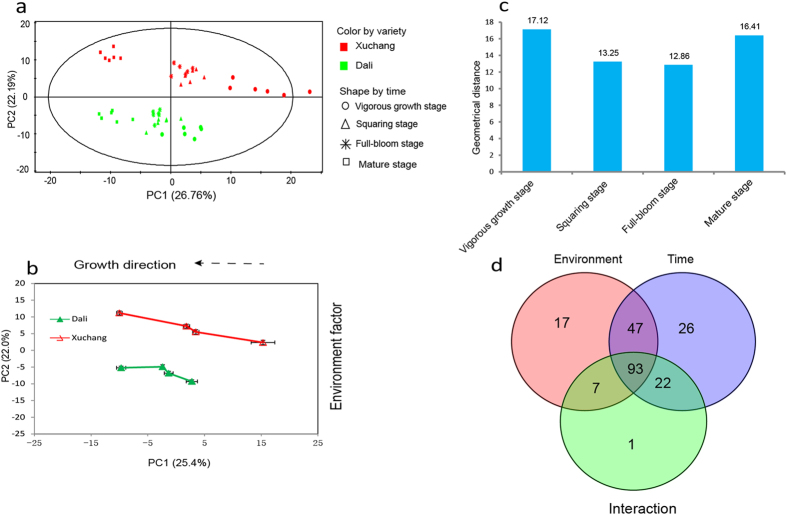
Time course of metabolic alterations in *cv.* K326 grown in Dali and Xuchang. (**a**) PCA score plot of *cv.* K326 from Xuchang (red) and Dali (green) along the first and second principal components. (**b**) Time-dependent metabolic trajectory of the 2D PCA score plot during four developmental stages. Error bars represent the standard error of measurement (SEM) at each time point. (**c**) Euclidean distance plot between the two growing locations at every development stage. (**d**) Venn diagram of two-way ANOVA defining the significantly different variables associated with planting areas (environment) and growth stage (time) and showing their interactions.

To clarify the metabolic characteristics associated with environment and growth, time-dependent metabolic responses to geographical regions were investigated. K326 is a flue-cured cultivar with wide adaptability to different regions^24^. The green leaves of K326 plants grown in Xuchang and Dali were collected. A total of 48 tobacco samples were collected during four growth periods, as shown in Supplementary Table S1. Four developmental stages, namely vigorous growth, squaring, full-bloom and middle-leaf mature stage, covered the growth and senescence of tobacco leaves. All of the samples separated into two clusters in the PCA score plot according to planting region (Fig. 2a). The corresponding PCA trajectory score plot revealed distinct metabolic fluctuations that were associated with growth and environmental factors along PC1 and PC2, respectively (Fig. 2b). The geometric distances between the two planting districts were used to evaluate the metabolomic changes in response to environmental influences across growth stages. Increased metabolic variations between the two planting regions were observed during the vigorous growth and ripening stages (Fig. 2c), suggesting that both environment (planting location) and growth (four growing periods) markedly impact metabolic phenotype. Two-way ANOVA analysis was performed to define the variables associated with location and growth. Totals of 17 and 26 metabolites (2-way ANOVA, *p* < 0.05) were markedly altered following environmental and growth-period changes, respectively (Fig. 2d). The metabolites significantly affected by planting region primarily included 7 organic acids, 3 nucleosides, 2 amino acids, 1 sugar derivative and 4 other metabolites. A hierarchical cluster analysis (HCA) of these metabolites was conducted to explore changes in the patterns between the two planting regions based on the Pearson correlation coefficients of metabolites. Two patterns, irrespective of growth stage, were observed (Supplementary Fig. S3). A majority of metabolites, except ethanolamine phosphate, showed higher levels in Xuchang during all four growth periods. Twenty-six metabolites, including fatty acids, organic acids, and amines, were greatly influenced by growth stage. Two growth tendencies (monotonous rising and falling) were observed in the HCA plot (Supplementary Fig. S4). Group 1, which includes inosine, cysteine and oxalic acid, showed a continuous increase from vigorous growth to maturity. In contrast, the metabolites of group 2, which includes fatty acids and amines, progressively decreased. Interestingly, the levels of these metabolites showed no significant differences between the two planting regions during the four growth periods.

**Figure 3:**
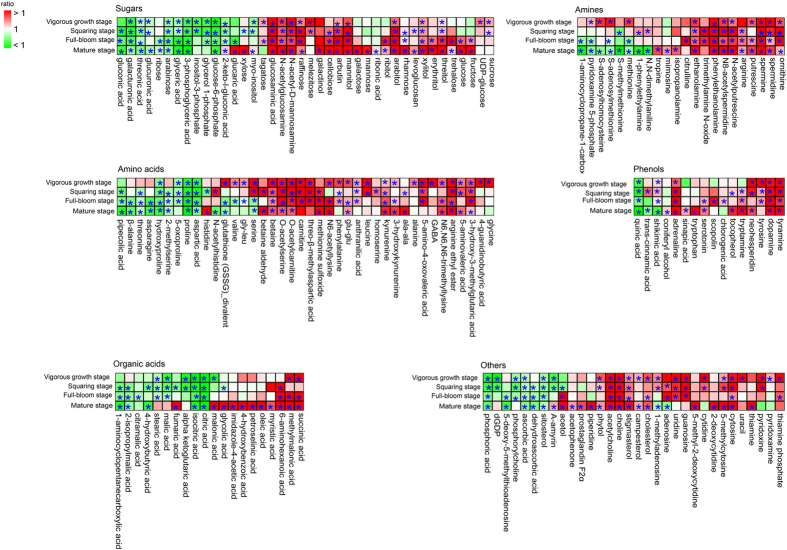
Heat map analysis of metabolites that differ between Dali and Xuchang. The ratio of Xuchang to Dali (Xuchang/Dali) for each metabolite was subjected to heat map analysis. Red and green indicate ratios of more than 1 and less than 1, respectively. *p < 0.05 between Xuchang and Dali.

After the removal of variables influenced by the growth period or by environmental factors, 170 metabolites that significantly differed (*p* < 0.05) in plants grown in Xuchang and Dali were identified using a non-parametric Mann-Whitney *U*-test. These metabolites were associated with environment-growth interactions. To visualize the time dependence of metabolic responses to the environment, heat maps were constructed that were based on metabolite categories (Fig. 3). Prior to heat map analysis, the metabolite ratio (Xuchang/Dali) was calculated. A majority of metabolites, including intermediates of sugar metabolism, amino acid metabolism, polyamine metabolism, phenol metabolism, fatty acid metabolism, alkaloid metabolism and vitamin metabolism, were more abundant in tobacco plants from Xuchang than in those from Dali. In contrast, intermediates in the TCA cycle, saccharic acid metabolism, asparagic acid and proline metabolism were more abundant in tobacco plants from Dali than in those from Xuchang. The significances of the correlation coefficients^25^ between content of metabolites and climatic factors (temperature, rainfall and sun exposure hours) were calculated to evaluate the effects of climate factors on differential metabolites (Fig. S5). Most of the metabolites are correlated with temperature and rainfall compared with sun exposure hours. The intermediates of sugar metabolism and shikimate-phenylpropanoid metabolism are positively correlated with temperature and negatively correlated with rainfall. In contrast, the intermediates of TCA cycle and glycolysis displayed clear negative correlations with temperature. Both of rainfall and temperature had negative effects on the content of most organic acids. The content of intermediates of methionine metabolism, amino acids from the TCA cycle showed positive correlations with rainfall and negative correlations with temperature. In contrast, clear positive correlations were observed between temperature and the content of amino acids from serine metabolism and intermediates of nucleotide and polyamines metabolisms.

## Discussion

**Figure 4:**
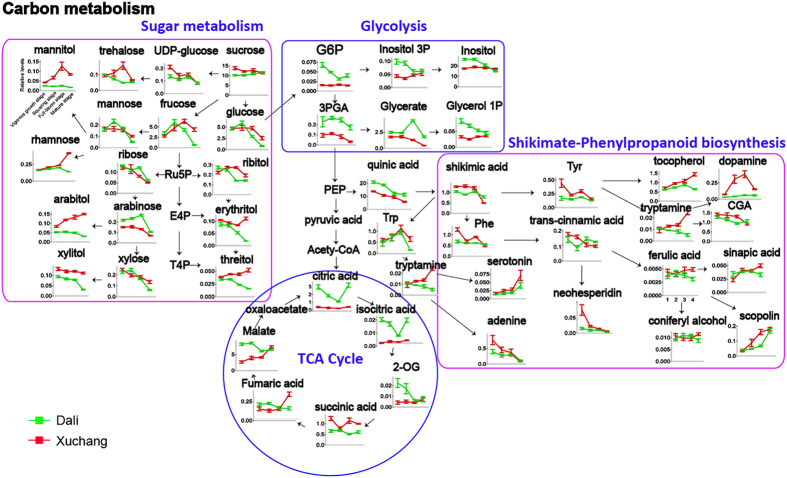
Pathway map of carbon metabolism-associated metabolites differing between Dali and Xuchang. Red and green lines indicate the relative abundances of metabolites in Xuchang and Dali, respectively. The following metabolites are abbreviated: glucose-6-phosphate (G6P), 3-phosphoglycerate (3PGA), inositol-3-phosphate (Inositol 3P), glycerol-1-phosphate (Glycerol 1P), ribulose-5-phosphate (Ru5P), erythritol-4-phosphate (E4P), threitol-4-phosphate (T4P), phosphoenolpyruvic acid (PEP), tryptophan (Trp), phenylalanine (Phe), tyrosine (Tyr), chlorogenic acid (CGA), and 2-oxoglutaric acid (2-OG). Each value represents the mean ± SEM.

**Figure 5:**
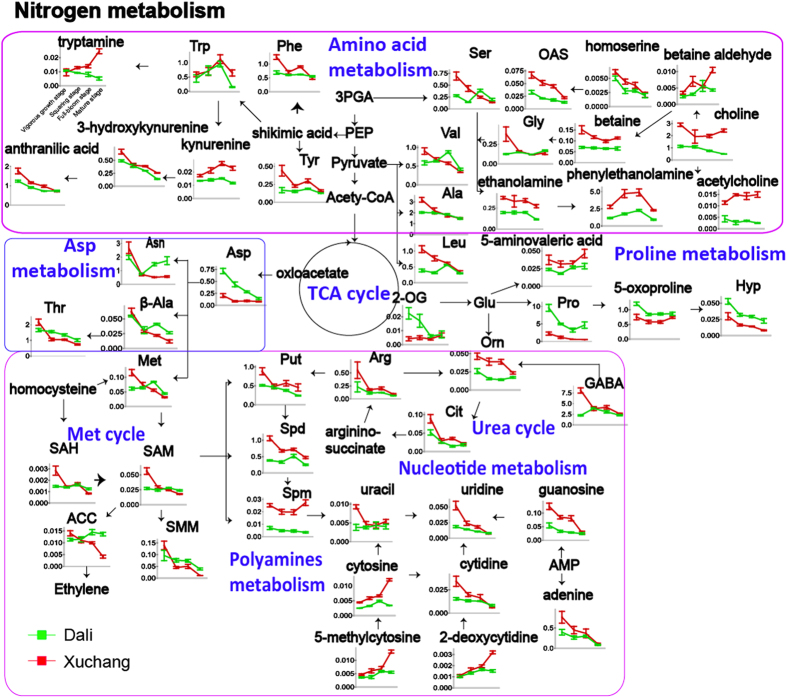
Pathway map of primarily nitrogen metabolism-associated metabolites differing between Dali and Xuchang. Red and green lines indicate the relative abundances of metabolites in Xuchang and Dali, respectively. The following metabolites are abbreviated: tryptophan (Trp), phenylalanine (Phe), tyrosine (Tyr), 3-phosphoglycerate (3PGA), phosphoenolpyruvic acid (PEP), serine (Ser), O-acetylserine (OAS), glycine (Gly), valine (Val), alanine (Ala), leucine (Leu), 2-oxoglutaric acid (2-OG), glutamic acid (Glu), proline (Pro), hydroxyproline (Hyp), ornithine (Orn), arginine (Arg), putrescine (Put), spermidine (Spd), spermine (Spm), citrulline (Cit), 4-aminobutyric acid (GABA), methionine (Met), S-adenosylhomocysteine (SAH), S-adenosylmethionine (SAM), 1-aminocyclopropane-1-carboxylic acid (ACC), S-methylmethionine (SMM), aspartic acid (Asp), asparagine (Asn), threonine (Thr), ß-alanine (ß-Ala), and adenosine monophosphate (AMP). All data are displayed as mean ± SEM.

Carbon (C) (Fig. 4) and nitrogen (N) metabolism (Fig. 5) represent intuitive parameters for comparing time-dependent metabolic fluctuations in tobacco leaves that are associated with environmental factors.

C metabolism (e.g., sugar metabolism, glycolysis, TCA cycle and shikimate-phenylpropanoid metabolism) is responsible for the production of accessible energy, resistance metabolites and carbon skeletons for plant life activities during growth and development^26^. C metabolism is closely related to plant growth and senescence, and it can be divided into C assimilation, C transformation and C accumulation among various plant physiological processes (e.g., photosynthesis and respiration). Glucose and fructose, two important reducing monosaccharides, are the degradation products of sucrose and starch. A sharp decline in glucose and fructose content was observed after the squaring stage in plants grown in Dali and at the full-bloom stage in plants grown in Xuchang, indicating that the shift from C assimilation to C accumulation occurred later in plants grown in Xuchang. Interestingly, sucrose, a cytoplasmic product produced during photosynthesis in plant cells by sucrose synthase, can be used as a transport sugar for entry into glycolysis and the TCA cycle for the production of ATP and NADH^27^. The high levels of sucrose observed in plants grown in Xuchang reflected higher rates of photosynthesis and higher energy demands in plant tissues. Additionally, increased levels of sugar alcohols (e.g., erythritol, threitol, xylitol and ribitol) derived from the pentosephosphate (PPP) pathway were also observed in plants grown in Xuchang, likely reflecting the higher respiratory rate of the PPP pathway in these plants. The marked accumulation of some sugar compounds (e.g., trehalose, mannitol and rhamnose) in plant samples obtained from Xuchang might also suggest that the polysaccharides of the plant cell wall were rapidly decomposed to produce small sugar molecules, such as osmolytes, to protect the cell membrane and plant proteins from high temperature or drought stress damage at the mature stage (Supplementary Fig. S2 and Fig. S5)^28^,^29^,^30^. Energy metabolism, principally comprising glycolysis and the TCA cycle, generates massive amounts of energy compounds (e.g., ATP and NADH) and precursors for various plant physiological processes through the oxidative decomposition of carbohydrates using a series of enzymes in the plant cell^31^. It was clear that lower amounts of the intermediates of central carbon metabolism (glycolysis and TCA cycle) were present in plants from Xuchang compared with those from Dali, a finding that is likely associated with increases in energy consumption^32^ and with transformation rates from C metabolism to N metabolism^33^. The shikimate-phenylpropanoid pathway generates numerous antioxidants (e.g., flavonoids, phenols and lignins) and their precursors (e.g., aromatic amino acids and shikimic acid) to scavenge or inhibit the synthesis of reactive oxygen species (ROS) in plant cells under biotic or abiotic stresses, thereby protecting cell proteins, membrane lipids, DNA and other cellular components from serious injury^34^. The increased content of defense-related metabolites (e.g., dopamine, chlorogenic acid, scopolin, and tocopherol) and their precursors (e.g., phenylalanine, shikimic acid and tryptamine) from shikimate-phenylpropanoid metabolism in the Xuchang plants could be closely associated with the high temperature and less abundant rainfall experienced during the mature stage (Supplementary Fig. S2).

These results show that the higher rate of photosynthesis, PPP metabolism and energy metabolism in Xuchang plants provides sufficient C-skeletons and energy for plant physiological metabolism. Furthermore, the metabolic shift from C assimilation to C accumulation was different between plants grown in Dali (after the squaring stage) and Xuchang (after the full-bloom stage) and likely reflected the ecological conditions of the growing regions. The higher levels of antioxidants detected in Xuchang plants relative to Dali plants suggest that the shikimate-phenylpropanoid pathway is more active to generate more defense-related metabolites to cope with the oxidative stress in Xuchang. The higher levels of intermediates of PPP metabolism (erythritol, arabitol and xylitol) and downstream shikimate-phenylpropanoid pathway compounds (tryptamine, dopamine and tocopherol) in Xuchang plants compared with Dali plants at the mature stage suggest that the respiration-related PPP pathway is enhanced in Xuchang plants, which might also be an important factor that underlies the metabolic differences observed between the two growing locations at the mature stage.

N metabolism, including amino acid metabolism, polyamine metabolism, the urea cycle and nucleotide metabolism, is a basic physiological mechanism for the synthesis and decomposition of nitrogenous compounds in plants^35^. Amino acids, as important energy metabolites and synthetic precursors of various bioactive molecules, are divided into aromatic, aliphatic and heterocyclic amino acid compounds based on chemical structures. Glycine and serine, two important photorespiration products, provide one-carbon (1-C) units that participate in various metabolic pathways, including nucleic acid metabolism, polyamine metabolism and betaine synthesis, through glycine decarboxylase (GDC)^36^ and serine hydroxymethyltransferase (SHMT)^37^. Notably, the higher glycine and serine levels in Xuchang plants could be attributed to their higher rates of photorespiration. Additionally, a greater abundance of photorespiration-associated intermediates of nucleoside metabolism, polyamine metabolism, the urea cycle, and betaine metabolism were observed in Xuchang plants, suggesting that higher photorespiration rates enhance the 1-C unit flux to N metabolism in Xuchang plants. Aromatic amino acids, as the biosynthetic precursors of some antioxidants, are upregulated to provide some osmoprotective functions in plant cells under heat and drought stress^32^. The levels of most aromatic amino acids (e.g., tyrosine, phenylalanine, kynurenine and 3-hydroxykynurenine) were higher in Xuchang plants, an effect likely associated with the higher temperature and dehydration stress in Xuchang plants at the mature stage (Supplementary Fig. S2). In contrast, an increased abundance of amino acids from the TCA cycle (e.g., proline, 5-oxoproline, hydroxyproline and aspartic acid) was observed in Dali plants compared with Xuchang plants. Cell wall hydroxyproline might be associated with cell elongation during plant growth. Extensin, a hydroxyproline-rich glycoprotein (HRGP), facilitates the extension of the plant cell wall and inhibits cell elongation^38^. Tobacco leaves grown in Xuchang exhibited longer leaf widths and lengths, lower heights, and thicker stem girths, suggesting that this extension protein might be highly expressed to restrain growth in Xuchang plants compared with Dali plants (Supplementary Table S2). All of the metabolites in the methionine (Met) cycle showed higher levels in Xuchang plants prior to the squaring stage but lower levels after squaring. Ethylene, as a vital downstream product of the Met cycle^39^, regulates the mature stage of plants^40^. Metabolites associated with ethylene in the Met cycle (e.g., S-adenosylmethionine, methionine, and 1-aminocyclopropane-1-carboxylate) likely induce higher levels of ethylene prior to the squaring stage and then lower levels of ethylene from the squaring to the mature stage in Xuchang plants. In accord with the metabolic changes in the ethylene precursor content during the growth stages, rapid tobacco growth is observed before the squaring stage in Xuchang plants and is followed by slow growth during later periods of development. In addition, nitrogenous compounds, including the intermediates of amino acid metabolism, the urea cycle, nucleotide metabolism and the Met cycle, show an overall decrease in the two planting regions, and differences in the metabolite content between Xuchang and Dali plants are gradually reduced as the plants mature, consistent with the metabolic responses of markers for primary nitrogen assimilation rates, including glutamine/2-oxoglutaric acid, malate/2-oxoglutaric acid and glutamine/glutamate^41^ (Supplementary Fig. S6). Significant differences in the nitrogen assimilation rates of tobacco plants grown in different locations were observed during the vigorous growth stage.

In summary, pseudotargeted GC-SIM-MS and CE-MS techniques were combined to investigate a time course of the metabolic responses of tobacco leaves to growing districts during growth. The complicated metabolic pattern was concerned with two planting districts over various physiological stages of leaf development from vigorous growing to squaring, full-bloom and mature stage during the leaf life cycle. The effects of both environment and growth conditions on metabolic phenotype of tobacco leaves were discussed. The results provided informative metabolic profiling data for further statistical analysis. Multivariate analysis illustrated that planting location has a much stronger effect than cultivar on the tobacco metabolic phenotype during plant growth and senescence. Significant correlations between the climatic factors and differential metabolites were observed, especially the temperature and rainfall during plant growth in the field. Furthermore, the links in metabolic changes of C and N pools in tobacco tightly associated with environmental and growth factors to physiological processes were comprehensively investigated. A comparison of tobacco plants grown in Dali and Xuchang showed that Xuchang plants exhibit higher rates of photosynthesis, photorespiration and respiration to supply sufficient energy and C-skeletons for the biosynthesis of N-containing metabolites. The shikimate-phenylpropanoid pathway was activated to generate an increased abundance of antioxidants to protect tobacco plants against oxidative stress in Xuchang.

## Methods

### Plant materials

A total of 143 fresh tobacco leaves, including 71 samples from Xuchang, Henan and 72 samples from Dali, Yunnan, were collected and used in this study (Supplementary Table S1). The Xuchang District in Henan province generate tobacco plants with strong flavors, and the Dali Districts in Yunnan province generate plants with delicate flavors (Fig. 1b)^42^. Three primary flue-cured tobacco cultivars, K326, ZY100 and HD, were investigated during four critical developmental stages (vigorous growing, squaring, full-bloom and mature stage). All samples were collected at approximately ten o’clock in the morning to avoid the effects of diurnal variations on the metabolic profiles. Five to six biological duplicates of each sample were collected and rapidly frozen in liquid nitrogen to inhibit the decline of enzyme activity in the plant tissue. Subsequently, the tobacco leaves were freeze-dried and ground to powder at a low temperature. After screening through an 80-mesh sieve, the tobacco powder was stored at –80 °C until further analysis. A quality-control (QC) sample was obtained after mixing equal amounts of each sample, and this sample was used to establish the pseudotargeted metabolomics method and to monitor the robustness of the analytical method. Climate information, including temperature (°C), rainfall (mm) and sunshine hours (h), was obtained from the local weather bureau (Supplementary Fig. S2).

### Sample preparation

Tobacco leaves were prepared for GC-MS analysis as previously described^2^. Briefly, a mixed solution of 4.15 μg/mL tridecanoic acid (internal standard) in isopropanol/acetonitrile/water (3:3:2, v/v/v) was prepared for metabolite extraction from the lyophilized tobacco leaf. Approximately 10 mg of tobacco powder was transferred to a 2-mL Eppendorf tube and immersed in 1.5 mL of extraction solvent. Subsequently, the solution was continuously vortexed for 4 min to destroy plant cells and extract metabolites. After centrifugation at 14,000 rpm for 10 min to remove insoluble material, 500 μL of the supernatant was vacuum-dried in a Labconco centrifugal concentrator and stored at −80 °C until further analysis. A derivatization method that includes oximation and silylation reactions was performed to increase the volatility of metabolites. The dried residue was redissolved in 100 μL of methoxyamine pyridine solution (20 mg/mL) and incubated for 90 min at 37 °C. Subsequently, an 80-μL aliquot of MSTFA was added to the mixture, which was then derivatized for 60 min in a 37 °C water bath. Prior to injection, the solution was centrifuged at 14,000 rpm for 5 min to remove precipitates, and about 160 μL of the supernatant was transferred to a glass vial for injection.

For CE-MS analysis, the tobacco leaves were prepared as follows. First, tobacco leaf powder (13 mg) was weighed in a 2-mL Eppendorf tube and soaked in 300 μL of methanol containing 50 μM of L-methionine sulfone and D-camphor-10-sulfonic acid as internal standards 1 (IS1) to standardize the metabolite intensity. To separate polar and nonpolar metabolites, 300 μL of chloroform and 1.2 mL of water were added to the tube, which was vortexed for 30 seconds before and after the addition of chloroform and water. A two-phase system was obtained after centrifugation at 9,000 × *g* for 5 min at 4 °C. To prevent large molecular impurities (e.g., proteins and chlorophyll) from adsorbing onto the surface of the capillary tube, 400 μL of the upper methanol-water phase was filtered through a 5-kDa ultrafiltration membrane (Millipore, USA) and centrifuged at 12,000 × *g* for 2 hours at 4 °C. Subsequently, the filtrate was freeze-dried in a Vacuum Concentrator (Labconco, USA). The dried tobacco sample was dissolved in 35 μL of Milli-Q water containing 50 μM 3-aminopyrrolidine dihydrochloride, N,N-diethyl-2-phenylacetamide, trimesic acidand 2-naphtol-3,6-disulfonic acid disodium salt as internal standards 2 (IS2) to adjust the migration time. Eventually, 10 μL of supernatant was placed into an injection vial with a conical insert for CE-TOF MS analysis.

All samples were randomly pretreated and analyzed to decrease errors derived from preparation and instrument analysis. Additionally, the QC samples were identically inserted into the analytical sequence to monitor the reproducibility of the analytical method.

### Instrumental analysis

The GC-MS experiment was conducted using a Shimadzu GC/MS QP 2010 Plus (Kyoto, Japan). One microliter of derivatization solution was separated on a DB-5 ms fused-silica capillary column (length = 30 m, ID = 0.25 mm, df = 0.25 μm) (Agilent Technologies). The split ratio was set to 10:1. High-purity helium (99.9995%, China) was employed as a carrier gas with a constant flow rate of 1.2 mL/min. The initial column temperature was 70 °C for 3 min and was then ramped at 5 °C/min to 310 °C for 5 min. The total running time for the GC-MS analysis was 56 min. The temperatures of the injector, interface and the ion source were maintained at 280 °C, 280 °C and 240 °C, respectively. Electron impact at 70 eV was used as the ionization mode. A 33–600-*m*/*z* mass scanning scope was used on full scan mode at an acquisition rate of 5 scan/sec. The detector voltage and solvent delay time were controlled at 0.92 kV and 5.24 min, respectively. A light diesel oil sample was analyzed in full scan mode to obtain the retention times of n-alkanes (C_10_–C_30_), which were used to calculate the Kovat’s retention index (RI) of the plant metabolites.

The pseudotargeted method based on GC-SIM-MS was established as previously described, with few modifications^2^,^12^,^18^,^19^. Briefly, the full-scan data of a doubled-concentration QC sample was converted to a netCDF file using the Shimadzu postrun workstation. The AMDIS (automated mass spectral deconvolution and identification system, NIST) and Leco ChromaTOF (USA) software programs were combined to analyze the netCDF data and achieve peak deconvolution and identification. The selection of characteristic ions was accomplished using various software programs (e.g., AMDIS, Chroma TOF and homemade software). Furthermore, the group information of metabolites was obtained according to the differences in the retention times of adjacent peaks. Ultimately, 329 features, which were further separated into 63 groups, were defined in the pseudotargeted quantitative and acquired methods.

The CE-MS analysis was performed on an Agilent G7100A CE system coupled to a G6224A TOF/MS with electrospray ionization (ESI). The CE separation of each reconstituted sample was conducted on a fused-silica capillary (length = 80 cm; ID = 50 μm) (Human Metabolome Technologies, Inc., Japan). The capillary temperature was set to 20 °C. A minichiller (Huber, Germany) was used to maintain the temperature of the sample tray below 5 °C. To achieve coupling of CE with MS, a coaxial sheath liquid interface was developed. A 50% methanol/water solution with 0.1 μM hexakis(2,2-difluoroethoxy)phosphazene were used as the sheath liquid at a constant flow rate of 1 mL/min via the column at a split ratio of 1:100. The specific parameters for cation mode and anion mode of the mass spectrometer were displayed in the Supporting Information.

### Data processing and statistical analysis

For the GC-SIM-MS method, a quantitative analysis table containing characteristic ions and retention time (RT) of detected metabolites was established for peak alignment based on the SIM scan table^2^. Prior to statistical analysis, the peak area of each metabolite in each sample was normalized to the internal standard (tridecanoic acid).

For CE-MS, as described in previous studies^21^,^43^,^44^, the raw MS data were imported into the Agilent MassHunter workstation software (B.04.00) for noise filtering, peak extraction, identification, smoothing and peak area integration. Additionally, MethodMarker software (HMT, Japan) was used to correct the migration time of metabolites. Metabolite identification was performed according to the accurate masses and adjusted migration times of standard chemicals in the HMT database. The matching windows for the *m*/*z* and migration time of a metabolite were adjusted to ±20 ppm and ±1.0 min, respectively. Subsequently, the integration of peak area was conducted using Agilent quantitative analysis software, and a peak table containing accurate mass, migration time and peak area was obtained. The peak area of every metabolite was divided by the internal standard for the following statistical processing.

Principal component analysis (PCA) accompanied by UV scaling^45^ was performed using the SIMCA-P 11.0 program (Umetrics, Sweden) to visualize the sample distributions. Two-way analysis of variance (ANOVA) was used to achieve the discrimination of two independent factors using MetaboAnalyst^46^ (http://www.metaboanalyst.ca/). Each metabolite with a significant difference (*p* < 0.05) was screened with a non-parametric Mann-Whitney *U*-test using SPSS (Statistical Package for the Social Sciences) 18.0 software. A heat map analysis was conducted with MeV.v.4.8.1 software^47^ to visualize the relationships and relative levels of metabolites. The alteration trends of metabolites were depicted using VANTED V2.1.0 software^48^. The correlation significance test was calculated using SPSS 18.0 software, and metabolic correlation network with climate factors was constructed using the Cytoscape V2.8.3 software.

Metabolite identification in GC-MS and CE-MS was conducted as previously described^2^,^43^. Briefly, a commercial library search (e.g., Mainlib, Wiley, and Fiehn) and standard validations using to mass spectra, RT and RI were used as qualitative methods to analyze the metabolites acquired using GC-MS. Moreover, the metabolites in CE-MS were identified based on accurate mass and migration time matching between the detected metabolites and standard chemicals in mass spectral libraries containing 500 reference standards (HMT, Japan).

## Additional Information

**How to cite this article**: Zhao, Y. *et al.* A metabolomics study delineating geographical location-associated primary metabolic changes in the leaves of growing tobacco plants by GC-MS and CE-MS. *Sci. Rep.* **5**, 16346; doi: 10.1038/srep16346 (2015).

## Supplementary Material

Supplementary Information
