# Supplementary material for: A metabolomics study delineating geographical location-associated primary metabolic changes in the leaves of growing tobacco plants by GC-MS and CE-MS
Source: Sci Rep. 2015 Nov 9;5:16346. doi: 10.1038/srep16346 (PMC4637841; doi:10.1038/srep16346)
Supplement: Supplementary Information [file srep16346-s1.doc]

**Supplementary materials**

**A metabolomics study delineating** **geographical location-associated primary metabolic changes** **in the leaves of growing tobacco plants by GC-MS and CE-MS**

*Yanni Zhao^1+^, Jieyu Zhao^1,2+^, Chunxia Zhao^1^**, Huina Zhou^3^, Yanli Li^1^, Junjie Zhang^1^, Lili Li^1^, Chunxiu Hu^1^, Wenzheng Li^4^, Xiaojun Peng^2^, Xin Lu^1^*, Fucheng Lin^3^, Guowang Xu^1^*

^1^Key Laboratory of Separation Science for Analytical Chemistry, Dalian Institute of Chemical Physics, Chinese Academy of Sciences, Dalian 116023, China

^2^State Key Laboratory of Fine Chemicals, Dalian University of Technology, Dalian 116023, China

^3^China Tobacco Gene Research Center, Zhengzhou Tobacco Research Institute of CNTC, Zhengzhou, 450001, China

^4^Yunnan Academy of Tobacco Agricultural Sciences and China Tobacco Breeding Research Center at Yunnan, Yuxi, 653100, China

* Prof. Dr. Xin Lu, e-mail: [luxin001@dicp.ac.cn](mailto:luxin001@dicp.ac.cn).

+ These authors contributed equally to this paper

**CE-TOF/MS Analysis**

For the cation mode, the injection volume was approximately 3 nL via pressure injection at 50 mbar for 3 s. Formic acid (1 M in water, pH = 1.8) was employed as a background electrolyte, and the positive separation voltage was 27 kV for 30 min. The flow rate and temperature of dry gas (nitrogen) were controlled at 7 L/min and 300 °C, respectively. The voltages for the capillary, fragmentor and skimmer were set as 4000, 108 and 50 V, respectively. The nebulizer gas (nitrogen) pressure was 7 psi, and the octapole radio frequency voltage (Oct RFV) was 650 V. A mass scan range of TOF-MS was set from 60-1000 *m*/*z* at an acquisition rate of 1.5 spectra/sec. Protonated compounds (i.e., [2MeOH + H] (*m*/*z* 66.0631) and (2,2-difluoroethoxy)phosphazene (*m*/*z* 622.0289)) were used to adjust the mass precision of TOF-MS in real time.

For the anion mode, ammonium acetate (50 mM in water, pH = 8.5) was used as the background electrolyte. Approximately 25 nL of reconstituted extract was injected at a pressure of 50 mbar for 25 sec. Positive voltage was maintained at 30 kV for 40 min. The mass spectrometer conditions included capillary voltage of 3500 V, fragmentor voltage of 125 V and scanning range of 50-1000 *m*/*z*. Two reference metabolites (i.e., [CH3COOH-H]^−^, *m*/*z* 60.0172) and [hexakis(2,2-difluoroethoxy)phosphazene + CH3COOH-H]^−^, *m*/*z* 680.03554) were used to adjust the mass precision of TOF-MS in real time. The other instrumental parameters were identical to those in cation mode.

**Table S1.** Sample Information

| Sample | Duplicates | Development Stage | Locations |
| --- | --- | --- | --- |
| HH2 | 6 | Vigorous growth stage | Xuchang, Henan |
| HH4 | 6 | Squaring stage | Xuchang, Henan |
| HH5 | 5 | Full-bloom stage | Xuchang, Henan |
| HH8 | 6 | Middle leaf mature stage | Xuchang, Henan |
| HZ2 | 6 | Vigorous growth stage | Xuchang, Henan |
| HZ4 | 6 | Squaring stage | Xuchang, Henan |
| HZ5 | 6 | Full-bloom stage | Xuchang, Henan |
| HZ8 | 6 | Middle leaf mature stage | Xuchang, Henan |
| HK2 | 6 | Vigorous growth stage | Xuchang, Henan |
| HK4 | 6 | Squaring stage | Xuchang, Henan |
| HK5 | 6 | Full-bloom stage | Xuchang, Henan |
| HK8 | 6 | Middle leaf mature stage | Xuchang, Henan |
| YH2 | 6 | Vigorous growth stage | Dali, Yunnan |
| YH4 | 6 | Squaring stage | Dali, Yunnan |
| YH5 | 6 | Full-bloom stage | Dali, Yunnan |
| YH8 | 6 | Middle leaf mature stage | Dali, Yunnan |
| YZ2 | 6 | Vigorous growth stage | Dali, Yunnan |
| YZ4 | 6 | Squaring stage | Dali, Yunnan |
| YZ5 | 6 | Full-bloom stage | Dali, Yunnan |
| YZ8 | 6 | Middle leaf mature stage | Dali, Yunnan |
| YK2 | 6 | Vigorous growth stage | Dali, Yunnan |
| YK4 | 6 | Squaring stage | Dali, Yunnan |
| YK5 | 6 | Full-bloom stage | Dali, Yunnan |
| YK8 | 6 | Middle leaf mature stage | Dali, Yunnan |

**Table S2.** Agronomic traits of *cv.* K326 (n=10) grown in Dali, Yunnan and Xuchang, Henan

| Cultivars | Plant height (cm) | Leaf number | Stem girth (cm) | Pitch line (cm) | Maximum leaf length (cm) | Maximum leaf width (cm) | Length-width ratio |
| --- | --- | --- | --- | --- | --- | --- | --- |
|  | mean ± SD | mean ± SD | mean ± SD | mean ± SD | mean ± SD | mean ± SD | mean ± SD |
| Dali | 122.10±6.30 | 22.90±0.74 | 8.00±0.94 | 4.10±0.30 | 58.10±3.41 | 22.10±1.45 | 2.64±0.24 |
| Xuchang | 99.20±2.53 | 26.50±0.85 | 10.95±0.50 | 3.75±0.11 | 63.10±2.33 | 29.80±0.63 | 2.12±0.08 |

SD: standard deviation


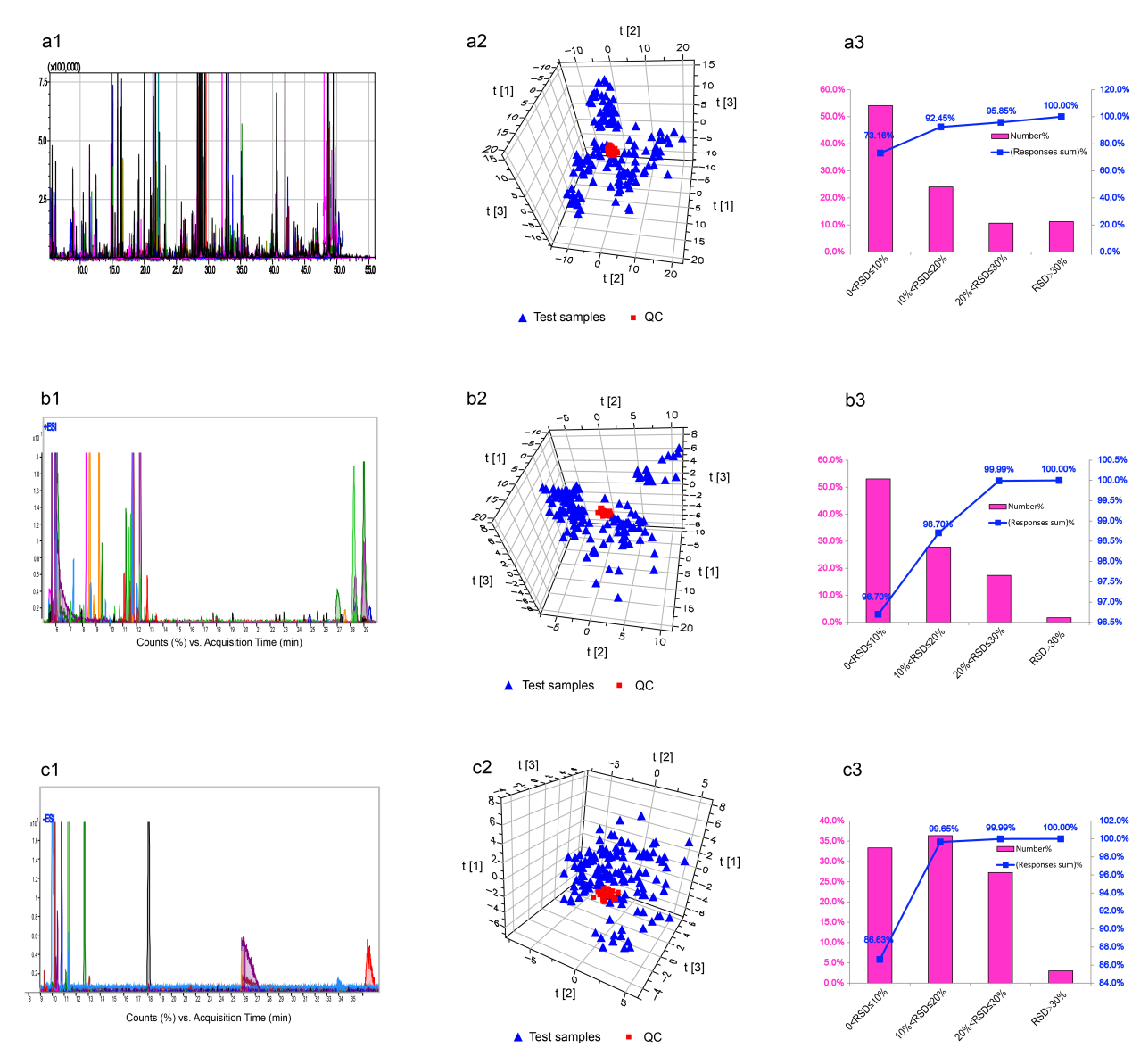


**Figure S1**. Analytical characteristics of the pseudotargeted GC-SIM-MS and CE-TOF-MS. a1, Pseudotargeted GC-SIM-MS chromatogram of the QC sample. b1 and c1, CE-MS extracted ion chromatograms of the QC samples using cationic mode (b1) and anionic mode (c1). a2, b2, and c2, PCA score plots for all samples analyzed by pseudotargeted GC-SIM-MS (a2) and by CE-MS using the cationic mode (b2) and the anionic mode (c2). a3, b3, and c3, RSD distribution plots of the metabolites in QC samples by pseudotargeted GC-SIM-MS (a3) and by CE-MS using the cationic mode (b3) and the anionic mode (c3).


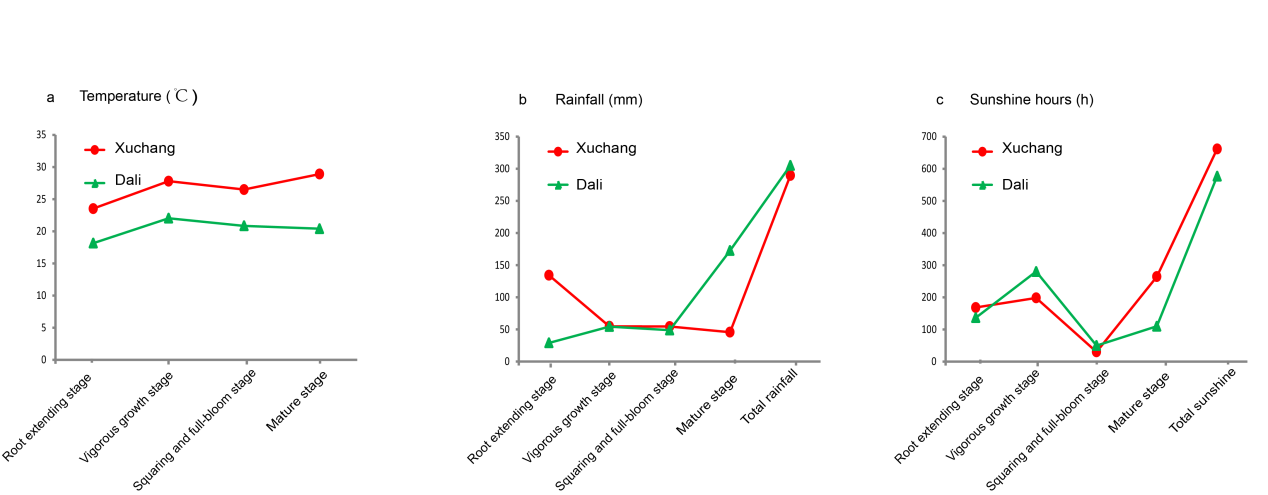


**Figure S2**. Climate information for Dali and Xuchang. a, Temperature (°C) of Dali and Xuchang. b, Rainfall (mm) of Dali and Xuchang. c, Sunshine hours (h) of Dali and Xuchang.


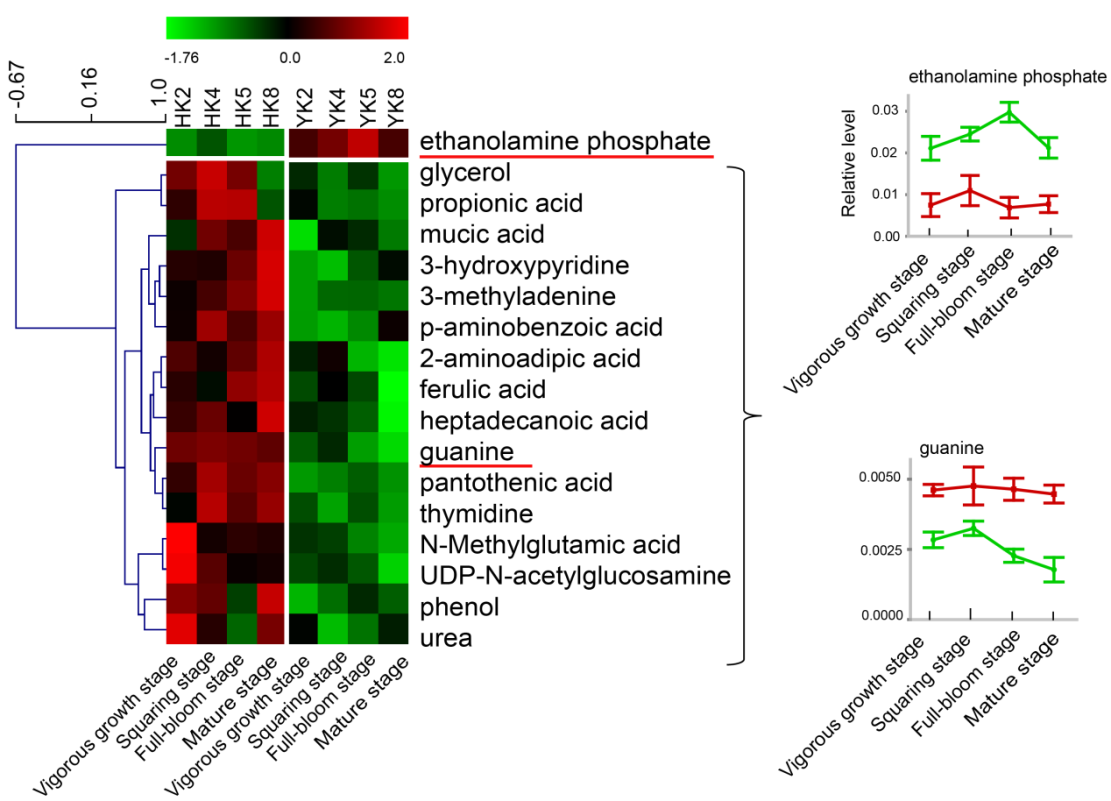


**Figure S3**. Heat map of differential metabolites associated with the planting locations and of variations of typical metabolites. The red and green lines in the variation tendency plots represent the metabolite abundances in Xuchang and Dali plants, respectively. Each value represents the mean ± SEM.


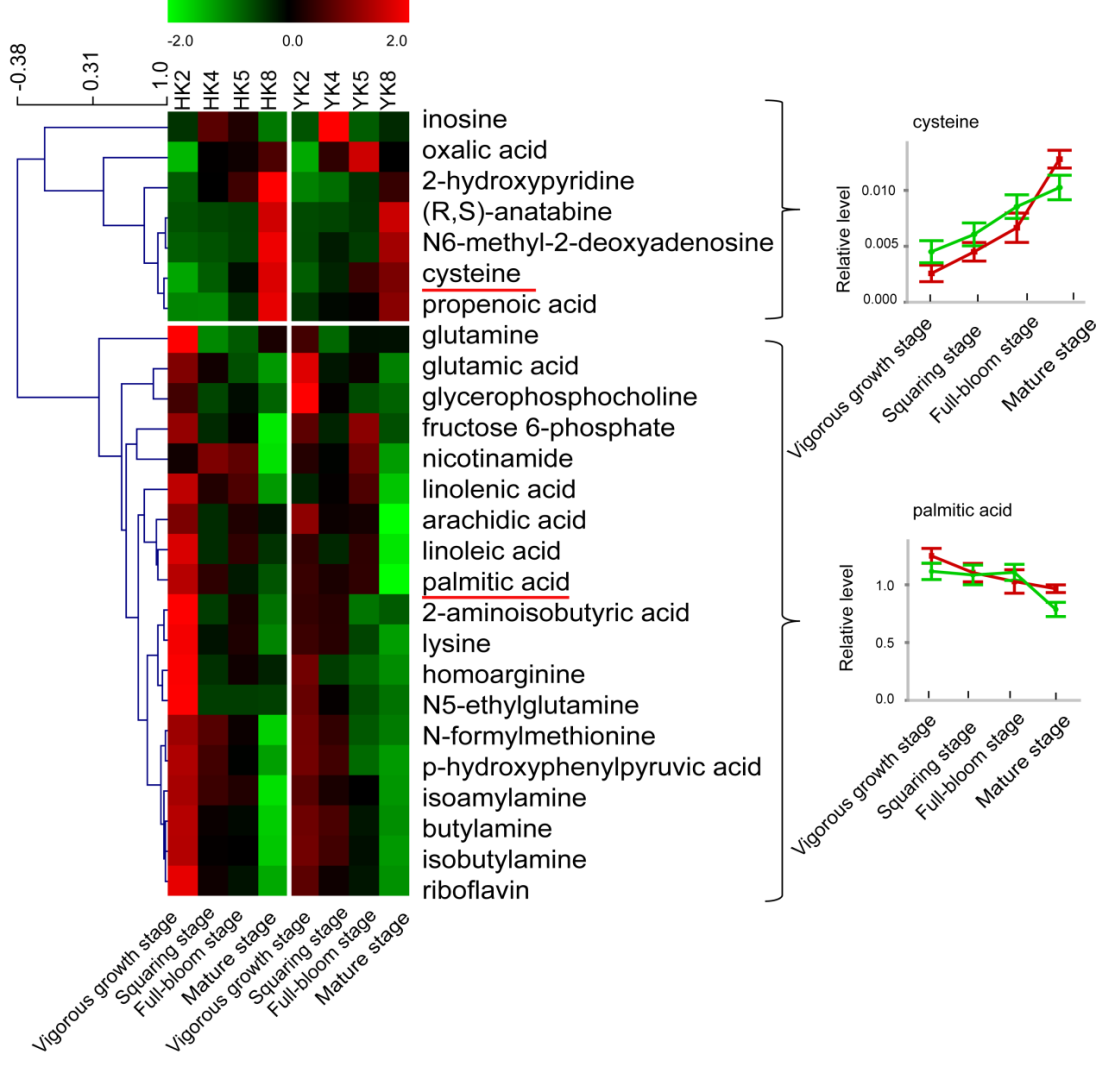


**Figure S4**. Clustering analysis of significantly different metabolites affected by developmental stage and of variations in typical metabolites. The red and green lines in the variation tendency plot represent the metabolite abundances in Xuchang and Dali plants, respectively. Each value is presented as mean ± SEM.


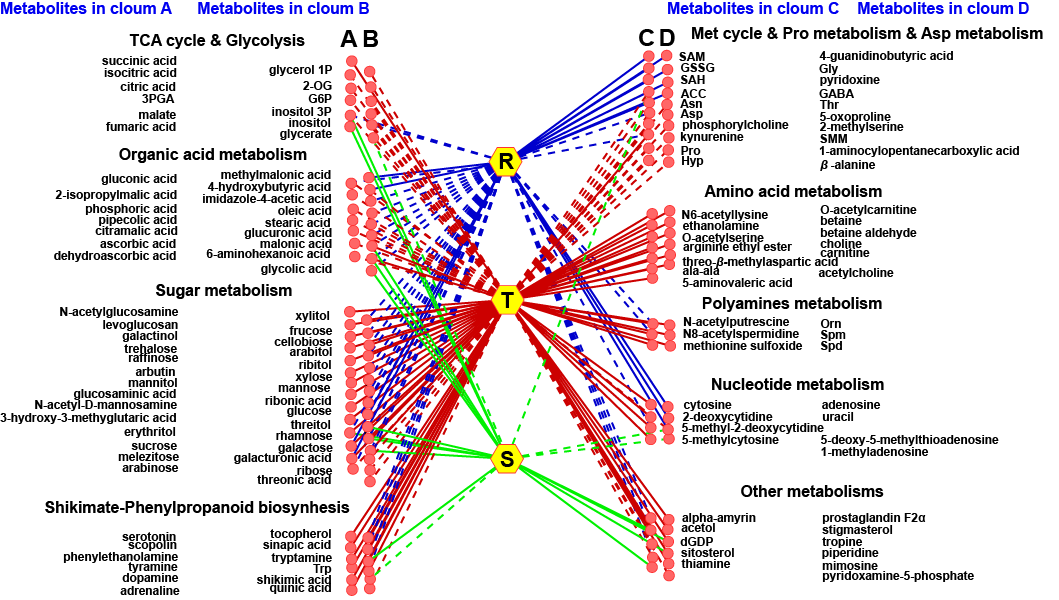


**Figure S5**. Correlation networks between differential metabolites and climatic factors. The *p*-value threshold of correlation significance test was fixed on 0.05. Solid and dotted lines represent positive and negative correlations, respectively. Red, blue and green lines represent the correlations with temperature (T), rainfall (R) and sunshine hours (S), respectively. The metabolites in columns A and B were the intermediates of carbon metabolism. The metabolites from nitrogen metabolism were showed in columns C and D. The following metabolites are abbreviated: glycerol-1-phosphate (Glycerol 1P), glucose-6-phosphate (G6P), 2-oxoglutaric acid (2-OG), 3-phosphoglycerate (3PGA), inositol-3-phosphate (Inositol 3P), tryptophan (Trp), S-adenosylhomocysteine (SAH), S-adenosylmethionine (SAM), 1-aminocyclopropane-1-carboxylic acid (ACC), S-methylmethionine (SMM), aspartic acid (Asp), asparagine (Asn), proline (Pro), hydroxyproline (Hyp), glycine (Gly), 4-aminobutyric acid (GABA), threonine (Thr), ornithine (Orn), spermidine (Spd), spermine (Spm), and glutathione oxidised (GSSG).


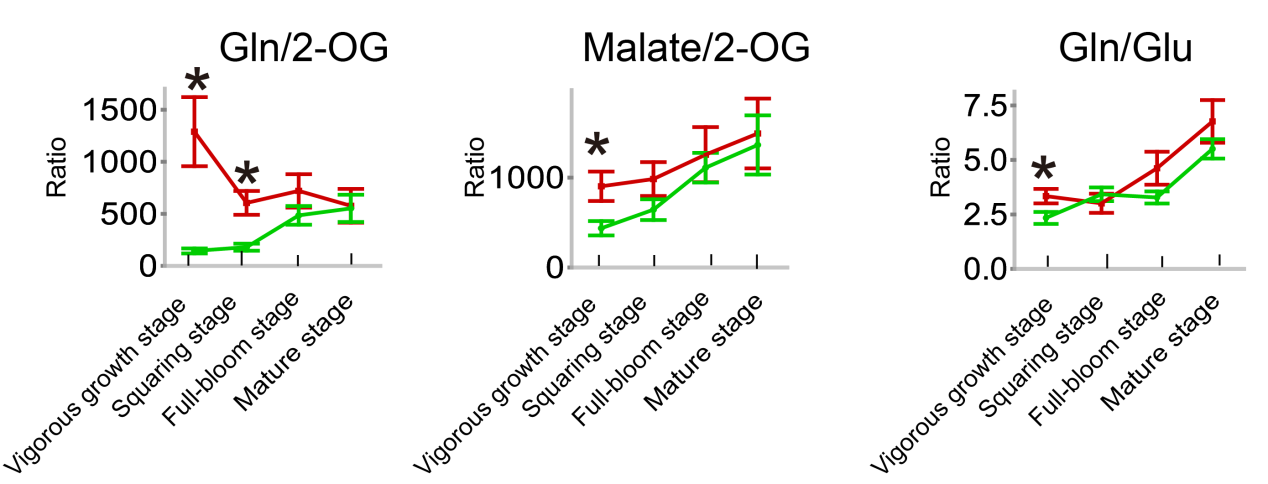


**Figure S6**. Relative ratios of metabolites in Xuchang and Dali plants during the four developmental stages. The red and green lines indicate Xuchang and Dali plants, respectively. * The mean *p*-value of the metabolite ratio was less than 0.05 between Dali and Xuchang. The following metabolites are abbreviated: glutamine (Gln), 2-oxoglutaric acid (2-OG), and glutamate (Glu). Each value represents the mean ± SEM.
